# Experimental BTV-3 and BTV-8 infection of *Culicoides sonorensis* biting midges

**DOI:** 10.1186/s13071-025-06883-z

**Published:** 2025-06-20

**Authors:** Sophie Zeiske, Franziska Sick, Helge Kampen, Bernd Hoffmann, Martin Beer, Kerstin Wernike

**Affiliations:** Friedrich-Loeffler-Institut, Federal Research Institute for Animal Health, Südufer 10, Greifswald - Insel Riems, Germany

**Keywords:** Orbivirus, Bluetongue virus, BTV-3, *Culicoides*, Biting midges, Vector, Vector competence, Experimental infection

## Abstract

**Background:**

Bluetongue virus (BTV) is the etiologic agent of a major infectious disease of livestock and is transmitted between its ruminant hosts by *Culicoides* biting midges. The first outbreak ever recorded in central Europe was caused by serotype BTV-8 and led to a major epidemic. In 2023, serotype BTV-3 emerged in the Netherlands and spread rapidly to neighbouring countries. Compared with the BTV-8 outbreak in 2006, the course of the BTV-3 epizootic is more severe, in regards to clinical signs and faster spread of the virus.

**Methods:**

To explore possible causes of the different epidemiologies, we performed laboratory infection experiments and compared the replication properties of BTV-8 and BTV-3 in *Culicoides sonorensis* biting midges.

**Results:**

Oral infection with BTV-3 resulted in a significantly higher viral load in the infected midges with demonstrated replication than BTV-8 infection.

**Conclusions:**

The higher viral load observed in midges with BTV-3 replication than in midges with BTV-8 replication may be a factor contributing to the observed faster outbreak progression of the current BTV-3 outbreak in comparison to the BTV-8 outbreak in 2006/2007.

**Graphical Abstract:**

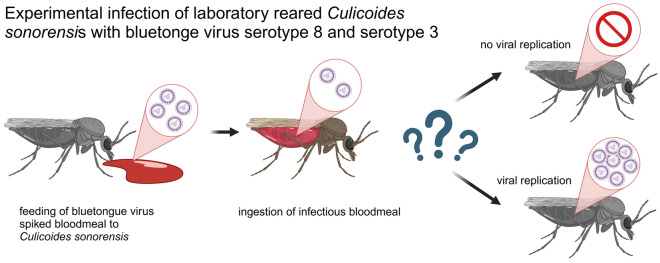

## Background

Bluetongue virus (BTV) is a non-contagious orbivirus transmitted primarily between its mammalian hosts by *Culicoides* biting midges, causing severe disease in ruminant livestock [1, 2]. The first BTV outbreak ever recorded in central Europe in 2006 was caused by a serotype 8 strain (BTV-8) and led to a major epidemic [3]. In 2023, a devastating BTV-3 outbreak started in the Netherlands and rapidly spread to neighbouring countries [4]. Compared with the clinical signs in domestic ruminants during the BTV-8 outbreak in 2006/2007, the clinical course of disease caused by BTV-3 is more severe [5]. BTV-3 induces high mortality in sheep and goats [6] and cattle [7]. Cases of calves with dullness, standing and sucking difficulties (“dummy calves”) caused by BTV-3 have been described in England, with similarity to BTV-8-affected calves [8].

Arthropod-borne pathogens such as BTV are mainly spread by the dispersal of infected vectors and the movement of infected livestock [4]. In Europe, the confirmed vectors of BTV with demonstrated replication in the salivary glands either from experimental infection studies or field-collected midges are *Culicoides imicola*, *Culicoides scoticus*, *Culicoides obsoletus*, *Culicoides nubeculosus*, *Culicoides impunctatus* and *Culicoides pulicaris* [9–14]. However, *C. nubeculosus* and *C. impunctatus* seem to play a minor role in the field [14, 15]. Additionally, *Culicoides chiopterus*, *Culicoides dewulfi* and *Culicoides punctatus* are considered competent vectors for BTV [14, 16, 17]. *Culicoides sonorensis* is considered the main vector for BTV in North America [18]. During vector monitoring in late 2023 in Germany near the Dutch border, BTV-3 was detected in a pool sample of *Culicoides* biting midges. The pool sample contained *C. obsoletus*, *C. scoticus* and *C. chiopterus* [19].

Comparison of the spread between farms of BTV-8 in 2006/2007 and BTV-3 in 2023 by transmission kernel analysis, which describes the distance-dependent probability of disease transmission from an infected farm to a susceptible farm, revealed a very similar kernel shape parameter of the BTV-8 and the BTV-3 outbreaks. This suggests that the mechanisms of disease spread through short-distance dispersal of infected midges and other modes for longer distances, such as livestock movement, were similar between the two outbreaks [4]. However, a much higher amplitude parameter was observed for the 2023 BTV-3 epidemic, indicating a faster disease spread. This could be due to higher temperatures of about 2 °C above normal during the observed period (September–November) of the 2023 BTV-3 outbreak compared with the corresponding period of the 2006 BTV-8 outbreak, since biting activity and virus replication in the biting midges are temperature-dependent [20].

The efficiency of virus transmission from host to midge is crucial for maintaining the natural infection cycle of BTV. While the efficiency of virus transmission from the infected midge to the vertebrate host is extremely high, only a few midges get infected after feeding on a viremic host, as demonstrated in experimental infection studies [21]. Next to the influence of higher temperatures, another reason for the observed faster disease spread of BTV-3 in 2023 than BTV-8 in 2006/2007 might be a higher infection and transmission efficiency of the midges for BTV-3 than for BTV-8 [4].

Laboratory colonies of the relevant European biting midge vector species are not available. Therefore, infection studies with BTV are commonly conducted by using field-caught midges or laboratory-reared *C. sonorensis.* The experimental infections studies investigating the vector competence of *Culicoides* species with different BTV strains conducted so far have been carried out with field-caught midges [9, 11, 22]. The laboratory colony of *C. sonorensis* is a suitable model to study infection dynamics under standardised laboratory conditions [20, 21, 23, 24] since this species plays a crucial role for BTV transmission in North America [18]. Although most experimental BTV-8 infection studies using field-captured midges aim to calculate replication rates, the results of different studies are not easily comparable due to differences in experimental design, sample processing and data analysis. They are also not easily comparable due to variation in the age structure of field-collected midges and uncertainty about conditions experienced by midges in the larval and adult stages prior to capture.

Apart from a study describing intrathoracic inoculation and oral infection of *C. sonorensis* with BTV-3 [25], no direct comparison of the currently circulating BTV-3 to BTV-8 from the 2006/2007 outbreak is available. Therefore, we performed infection experiments with a laboratory colony of *C. sonorensis* to directly compare the replication properties of BTV-8 and BTV-3 in biting midges.

## Methods

### Viruses

One BTV-3 isolate and one BTV-8 isolate were selected. BTV-8 strain BH311/06 was isolated from a German sheep during the 2006 outbreak on ENT-R cells and passaged two more times on ENT-R cells (RIE0455, Collection of cell lines in veterinary medicine [CCLV], Friedrich-Loeffler-Institut [FLI], Greifswald-Insel Riems, Germany) and 11 times on BHK-21 [C-13] cells (RIE0179, CCLV). BTV-3 was isolated from a German sheep in 2023 on KC cells (RIE1062, CCLV) and passaged twice on BHK21 (RIE164, CCLV) [19]. Both virus stocks were propagated on BHK21 cells (RIE164, CCLV).

### Experimental setup

**Fig. 1:**
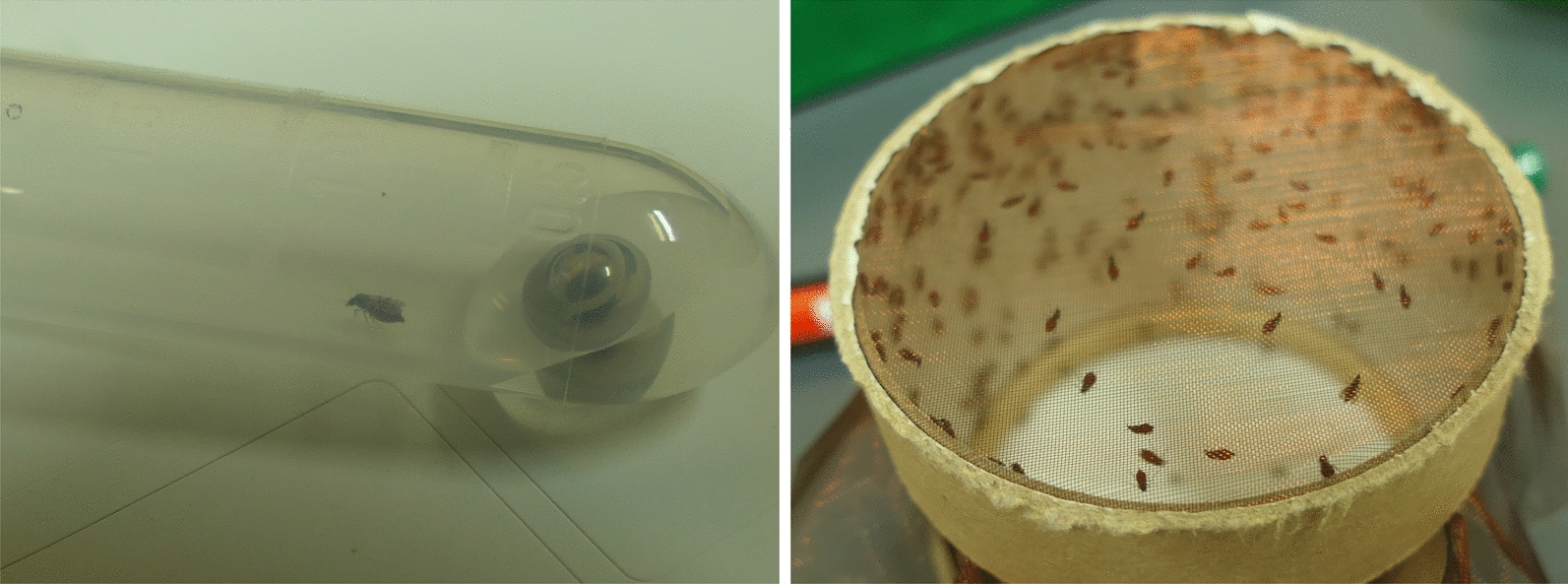
Freshly engorged *C. sonorensis* (left). The biting midge was placed individually in a tube containing 200 µL phosphate-buffered saline (PBS) and a 5 mm stainless steel bead for further processing. Engorged females in a new netted cardboard cage after blood feeding (right)

The laboratory colony of *Culicoides sonorensis* was developed and supplied by The Pirbright Institute [26]. This colony was reared in the biosafety level 2 insectary of the FLI, Greifswald-Insel Riems, as described previously [27]. The 3-day-old biting midges were offered caprine (trial 1) or ovine (trial 2) heparin blood, obtained from the FLI, mixed 1:1 with BTV-8 or BTV-3 in cell culture medium (Minimum Essential Medium). The blood meal contained 10^6^ 50% tissue culture infective dose per mL (TCID_50_/mL), which was confirmed by back-titration after feeding. As a negative control (NC), blood was mixed with virus-free cell culture medium. After preheating to 37 °C, the blood meal was offered to the midges using a “Hemotek membrane feeding system” (Hemotek, Blackburn, UK) for 30 min. Midges were sorted under short-term CO_2_ anaesthesia on a cooling plate. Clearly engorged females were transferred to a new cage and kept for the course of the experiment. Sixteen blood-fed midges per group (BTV-8, BTV-3, NC) were processed immediately after feeding (Fig. 1). The remaining blood-fed midges were kept inside gauze-covered cages in an incubator at 27 °C and a relative humidity of 85% with an 8 h dark/16 h light regime and supplied with 5% glucose ad libitum. After an incubation period of 6 days, the surviving midges were harvested. All midges were placed individually in tubes containing 200 µL phosphate-buffered saline (PBS) and a 5 mm stainless steel ball (Fig. 1). Two consecutive trials with the same set-up were performed as biological replicates and to achieve a higher number of analysable midges.

Following homogenization using a TissueLyzer (Qiagen, Hilden, Germany) for 3 min at 30 Hz, total RNA was extracted for each sample using the King Fisher 96 Flex (Thermo Scientific, Braunschweig, Germany) in combination with the NucleoMag VET kit (Macherey Nagel, Düren, Germany) according to the manufacturer’s instructions.

The RNA extracts were analysed by a BTV-specific reverse transcriptase quantitative polymerase chain reaction (RT-qPCR) [28] with an external full virus BTV-3 standard, which was used to calculate the number of BTV genome copies per midge. The BTV-3 standard is suitable for calculation of the genome copy numbers of both BTV-3- and BTV-8-infected midges, as the applied BTV-specific RT-qPCR cannot differentiate between the serotypes. The highest copy number detected in a midge of each day-0 group was used as a cut-off value for the virus intake background. Virus replication in midges of the day-6 group was assessed using the day-0 cut-off value. Midges exceeding the BTV copy number of the day-0 cut-off were considered replicating the virus. Midges with BTV copy number values below the cut-off were considered to contain residual blood meal without replication of the virus. The usage of this method was described in other vector competence studies in midges on BTV [29] and bovine ephemeral fever virus [30].

### Statistical analysis

Replication properties of midges were assessed as follows: Percentages of midges above the day-0 cut-off (midges with virus replication) were compared by two-sided Fisher exact test, and differences were regarded significant when *p* < 0.05. From the BTV genome copy load of the midges with virus replication, the corresponding day-0 cut-off value was subtracted, and the resulting values were compared using a Mann–Whitney test. Differences were regarded as significant when *p* < 0.05.

## Results and discussion

A total of 503 (first trial BTV-3), 485 (second trial BTV-3), 551 (first trial BTV-8) and 355 (second trial BTV-8) midges fed on the virus-spiked blood meal. A total of 16 midges per group and trial were processed immediately, and the remaining midges were transferred to a new cage for further incubation. Ingestion of virus-spiked blood (BTV-8 and BTV-3) led to polymerase chain reaction (PCR) positivity in all midges harvested directly after the blood meal (day 0). The highest copy numbers of BTV-3-infected midges from the day-0 group (day-0 cut-off values) in trial 1 and trial 2 were 59,040 BTV copies/midge and 159,000 BTV copies/midge, respectively. For the day-0 group of BTV-8-infected midges 313,200 BTV copies/midge for trial 1 and 366,800 copies/midge for trial 2 were set as the day-0 cut-off values. Out of all 1830 blood-fed midges that were kept for the 6-day incubation period, a total of 1121 (61.25%) of the midges survived.

**Fig. 2:**
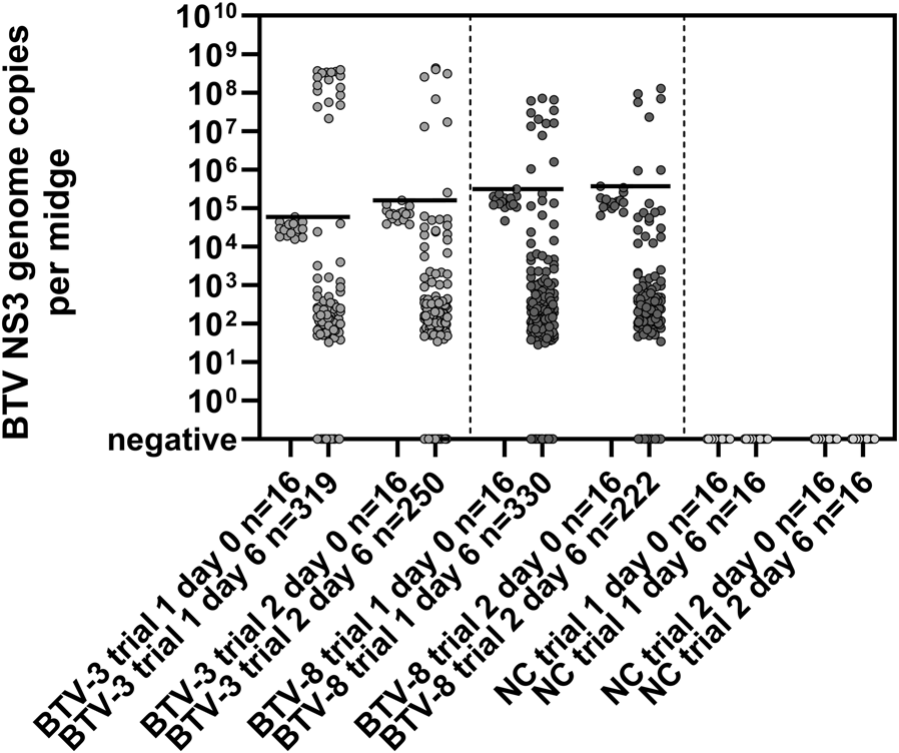
RT-qPCR results of midges experimentally infected with BTV-3- or BTV-8-spiked blood meals and midges fed with virus-free blood (negative control [NC]). Individual midges were tested for BTV genome immediately after ingestion of the blood meal (day 0) or 6 days after the blood meal (day 6). Horizontal black lines indicate the highest BTV copy number measured in any of the midges of the respective group immediately after blood meal ingestion. The experiment was performed in two subsequent trials, the trial number is given in the label of the *x*-axis

In the first trial using BTV-3, 89 out of 319 surviving midges tested positive by RT-qPCR at 6 days of incubation, and 17 of these (5.32%) had viral loads higher than the day-0 group, indicating efficient virus replication. In the BTV-8 group of the first trial, 197 out of 330 surviving midges tested positive by RT-qPCR, and 12 of them (3.64%) showed efficient virus replication. In the second trial, 133 out of 250 surviving midges of the BTV-3 group tested positive by RT-qPCR, and 8 of them (3.20%) showed virus replication. In the BTV-8 group of the second trial, 110 out of 222 surviving midges tested positive by RT-qPCR, and 7 (3.15%) of them showed virus replication. Midges of the negative control group tested negative by RT-qPCR at all times (Table 1; Fig. 2). Midges with BTV copy number values below the day-0 cut-off were considered to contain residual blood meal without replication of the virus. Midges exceeding the BTV copy number of the day-0 cut-off were considered replicating. Overall, 4.39% of the midges infected with BTV-3 replicated the virus, while 3.44% of the midges infected with BTV-8 replicated the virus (Fig. 2). The detailed results of the genome quantification are listed in the Supplementary Dataset in the Zenodo repository (Doi: 10.5281/zenodo.14888759 ) .

**Table 1:** Number of midges harvested at day 6 post infection

		Day-6 group	Number and percentage of BTV-RNA-positive midges	Number and percentage of midges with virus replication
Trial 1	BTV-3	319	89 (27.90%)	17 (5.32%)
	BTV-8	330	197 (59.70%)	12 (3.64%)
	NC	16	0	0
Trial 2	BTV-3	250	133 (53.20%)	8 (3.20%)
	BTV-8	222	110 (49.55%)	7 (3.15%)
	NC	16	0	0

All midges were analysed by BTV PCR. BTV-RNA-positive midges/midges with virus replication are indicated in absolute numbers and in percentage in brackets

The calculated rate of midges replicating BTV-8 presented in this study is in line with infection rates previously published on BTV-8 in *C. sonorensis*, ranging from 1.9% to 7.3% [21]. For BTV-3, an infection rate of 6.82% was described [25]. However, the experimental setup of these studies differed from our study in regard to incubation time and temperature, feeding method and analysis of the infection rate.

Statistical analysis showed that the differences in the percentages of midges with BTV-3 or BTV-8 replication are not significant (two-sided Fisher exact test, *p* = 0.4446; odds ratio [OR], 1.289; 95% confidence interval [CI], 0.7–2.4). However, a comparison of the BTV genome copy numbers of the midges replicating BTV-3 or BTV-8 showed a significantly higher genome load in midges replicating BTV-3 (Mann–Whitney test, *p* = 0.0001). Therefore, BTV-3 seems to have better replication properties compared with BTV-8 under controlled experimental conditions. The higher viral genome loads in the BTV-3-infected midges might have a functional meaning. An important replication barrier in midges that prevents full dissemination to the salivary glands is the midgut barrier. Midges in which the midgut barrier has been overcome exhibit high virus titres (> 2.5 log_10_ TCID_50_/mL). In cases in which infections are restricted to the cells of the mesenteron, lower titres are observed [31]. Although we did not perform virus titration of the midges, the higher genome copy numbers per midge in the BTV-3-infected group may suggest a more effective escape of the midgut barrier and full virus dissemination within the midge.

However, since virus replication in the biting midges is temperature-dependent, the climatic conditions should also be taken into account, both in the field and in experimental infection studies [20]. Besides the potential better replication properties of BTV-3 in midges, a temperature difference of about 2 °C between the 2023 BTV-3 outbreak and 2006 BTV-8 outbreak may be another factor contributing to the observed faster outbreak progression of BTV-3 in nature [4]. To ensure that the observed differences in replication are not solely driven by the high temperature used in the presented experimental infection study, further infections of midges should be conducted at other incubation temperatures in the future.

## Conclusions

Oral BTV-3 or BTV-8 infection of laboratory-reared *C. sonorensis* resulted in a significantly higher BTV genome load of virus-positive midges with demonstrated replication for BTV-3 than for BTV-8. The significantly higher BTV genome load may be a factor contributing to the observed faster outbreak progression of the current BTV-3 outbreak in comparison to the BTV-8 outbreak in 2006/2007. However, future experiments will have to evaluate dissemination and transmission rates of the different serotypes to confirm this result.

## Data Availability

The datasets generated and analysed during the current study are available in the Zenodo repository (doi: 10.5281/zenodo.14888759 ) .

## Abbreviations

BTV: Bluetongue virus
*C. sonorensis*: *Culicoides sonorensis*
PCR: Polymerase chain reaction
RT-qPCR: Reverse transcriptase quantitative PCR
NC: Negative control
TCID: Tissue culture infective dose
CCLV: Collection of cell lines in veterinary medicine
PBS: Phosphate-buffered saline
